# Coral micro- and macro-morphological skeletal properties in response to life-long acclimatization at CO_2_ vents in Papua New Guinea

**DOI:** 10.1038/s41598-021-98976-9

**Published:** 2021-10-07

**Authors:** Fiorella Prada, Leonardo Brizi, Silvia Franzellitti, Stefano Mengoli, Simona Fermani, Iryna Polishchuk, Nicola Baraldi, Francesco Ricci, Quinzia Palazzo, Erik Caroselli, Boaz Pokroy, Loris Giorgini, Zvy Dubinsky, Paola Fantazzini, Giuseppe Falini, Stefano Goffredo, Katharina E. Fabricius

**Affiliations:** 1 Marine Science Group, Department of Biological, Geological and Environmental Sciences, University of Bologna, Via F. Selmi 3, 40126 Bologna, Italy; 2 Department of Physics and Astronomy, University of Bologna, Viale Berti Pichat 6/2, 40127 Bologna, Italy; 3 Animal and Environmental Physiology Laboratory, Department of Biological, Geological and Environmental Sciences, University of Bologna, via S. Alberto 163, 48123 Ravenna, Italy; 4 Department of Management, University of Bologna, Via Capo di Lucca 34, 40126 Bologna, Italy; 5 Department of Chemistry ‘Giacomo Ciamician’, University of Bologna, Via F. Selmi 2, 40126 Bologna, Italy; 6 Department of Material Sciences and Engineering and the Russell Berrie Nanotechnology Institute, Technion – Israel Institute of Technology, Haifa, Israel; 7 School of BioSciences, University of Melbourne, Parkville, 3010 Australia; 8 Department of Industrial Chemistry “Toso Montanari”, University of Bologna, Viale Risorgimento 4, 40136 Bologna, Italy; 9 Interdepartmental Center for Industrial Research on Advanced Applications in Mechanical Engineering and Materials Technology, CIRI-MAM, University of Bologna, Viale Risorgimento 2, 40136 Bologna, Italy; 10 The Mina and Everard Goodman Faculty of Life Sciences, Bar-Ilan University, 52900 Ramat-Gan, Israel; 11 Australian Institute of Marine Science, PMB 3, Townsville, QLD 4810 Australia; 12 Fano Marine Center, The Inter-Institute Center for Research on Marine Biodiversity, Resources and Biotechnologies, Viale Adriatico 1/N, 61032 Fano, Italy; 13 Consiglio Nazionale delle Ricerche, Istituto per lo Studio dei Materiali Nanostrutturati (CNR-ISMN), Via P. Gobetti 101, 40129 Bologna, Italy

**Keywords:** Climate-change ecology, Ecology, Ecology

## Abstract

This study investigates the effects of long-term exposure to OA on skeletal parameters of four tropical zooxanthellate corals naturally living at CO_2_ seeps and adjacent control sites from two locations (Dobu and Upa Upasina) in the Papua New Guinea underwater volcanic vent system. The seeps are characterized by seawater pH values ranging from 8.0 to about 7.7. The skeletal porosity of *Galaxea fascicularis*, *Acropora millepora*, massive *Porites*, and *Pocillopora damicornis* was higher (up to ~ 40%, depending on the species) at the seep sites compared to the control sites. *Pocillopora damicornis* also showed a decrease of micro-density (up to ~ 7%). Thus, further investigations conducted on this species showed an increase of the volume fraction of the larger pores (up to ~ 7%), a decrease of the intraskeletal organic matrix content (up to ~ 15%), and an increase of the intraskeletal water content (up to ~ 59%) at the seep sites. The organic matrix related strain and crystallite size did not vary between seep and control sites. This multi-species study showed a common phenotypic response among different zooxanthellate corals subjected to the same environmental pressures, leading to the development of a more porous skeletal phenotype under OA.

## Introduction

Tropical coral reefs support the livelihoods of hundreds of millions of people around the world, harbor 25% of all marine species, and protect thousands of kilometers of shoreline from waves and storms^1^. However, coral reefs face an intensifying array of threats deriving from pollution and overexploitation which is leading to a decline in their health^2^. In addition, global climate change compounds these threats in multiple ways. Increases in seawater CO_2_ and associated decreases in carbonate ion concentration, known as ocean acidification (OA), are projected to have profound implications for marine calcifiers, as carbonate ions are essential for biotic calcification^3^. Coral responses to OA may be affected by several factors including colony morphology, size, skeletal mineralogy and structure, tissue thickness, symbiont types, and/or the mechanisms of nutrient acquisition^4^. Moreover, the discrepancy among responses could derive from different experimental designs and analytical methods (e.g., addition of acid vs CO_2_ bubbling to mimic OA), co-limiting environmental conditions (e.g., temperature, light intensity, flow, feeding, etc.), and exposure times (days to months or even life times)^5^.

To date, most studies both under controlled conditions and under natural conditions in the field (e.g., CO_2_ vents), support predictions of decreased rates of calcification and increased rates of dissolution and bioerosion as seawater pH decreases^6^. However, studies conducted using skeletal cores have shown that coral calcification rates have not declined at a constant rate as ocean pH decreased and temperatures warmed throughout the twentieth century. On the contrary, at some locations, calcification rates have remained stable and in others they have even increased over this time period^7–9^. Even where declines in calcification have occurred, many other factors such as ocean warming, sea level rise, changes in surface ocean productivity, as well as many localized anthropogenic disturbances co-occur with OA. These additional factors also influence coral growth and could obscure our ability to attribute changes in coral calcification solely to OA^10^.

Most of the available knowledge about OA effects on marine organisms derives from short-term laboratory or mesocosm experiments on isolated organisms^11^, which can substantially underestimate full organism acclimatization^12^. In fact, taxa that appear unaffected by high CO_2_ under controlled conditions may be: (1) vulnerable in the long-term^13^, (2) affected during life stages that were not considered during the experiment^14^, or (3) be indirectly affected by OA-driven ecological changes (e.g., food webs, competition, diseases and/or community structures, habitat properties such as microbial surface biofilms)^15^. Likewise, other taxa that respond negatively to OA under controlled conditions may be capable of acclimatizing in the longer term. Thus, field experiments, where organisms are naturally exposed to OA for their entire life, as found around submarine CO_2_ vents, could provide important new insights. However, vent systems are not perfect predictors of future ocean ecology owing to temporal variability in pH, spatial proximity of populations unaffected by acidification, and the unknown effects of other changing parameters (e.g., temperature, currents)^16^. Nonetheless, vents acidify sea water on sufficiently large spatial and temporal scales to integrate ecosystem processes such as reproduction, competition and predation^17^. Field-based studies conducted at volcanic CO_2_ seeps in Italy^17–19^, Japan^20^, Mexico^21^, and Papua New Guinea (PNG)^15^ provide a unique opportunity to investigate long-term effects of OA on marine ecosystems that have been naturally exposed to chronic low pH and concomitant altered carbonate chemistry parameters for years/decades. These studies have already demonstrated substantial changes in community structure and functional biodiversity^22^ of benthic species, as well as an array of responses to OA spanning from sharp decrease to no effect on calcification rate^23^.

Studies conducted on corals at volcanic CO_2_ vents in Papua New Guinea (PNG) have supported the mixed effects observed in laboratory experiments^15,24^. Hard coral cover is similar at acidified and control sites (33% versus 31%). However, the cover of massive *Porites* is doubled under OA, whereas the cover of more structurally complex corals is reduced by one third^24^. Some species are significantly less common or even absent under OA. For instance, while the coverage of *Pocillopora damicornis* decreases by 43% in acidified sites, in situ growth measurements have found small differences in linear extension rate^15^, but large differences in recruitment success^25^. Population reductions in situ, combined with observations of negative physiological impacts, including declines in calcification under OA, strongly suggest that low pH imposes selection pressure on less resilient taxa within the PNG system^23^.

The aim of this study was to assess the effects of long-term exposure to OA on the skeletal parameters (micro-density, porosity, bulk density) of four tropical zooxanthellate coral species *Galaxea fascicularis* (Linnaeus, 1767), *Acropora millepora* (Ehrenberg, 1834), massive *Porites* Link, 1807, and *P. damicornis* (Linnaeus, 1758), living at CO_2_ vents and adjacent control sites in Milne Bay Province, PNG^15^. Additional macroscale and microscale skeletal analyses, namely Time-Domain Nuclear Magnetic Resonance (TD-NMR), Thermogravimetric Analysis (TGA), and synchrotron high-resolution powder X-ray diffraction (HRPXRD) analyses were performed on *P. damicornis*, the only species displaying differences in micro-density at the seep sites compared to control.

## Materials and methods

### Study sites and coral sampling

**Figure 1:**
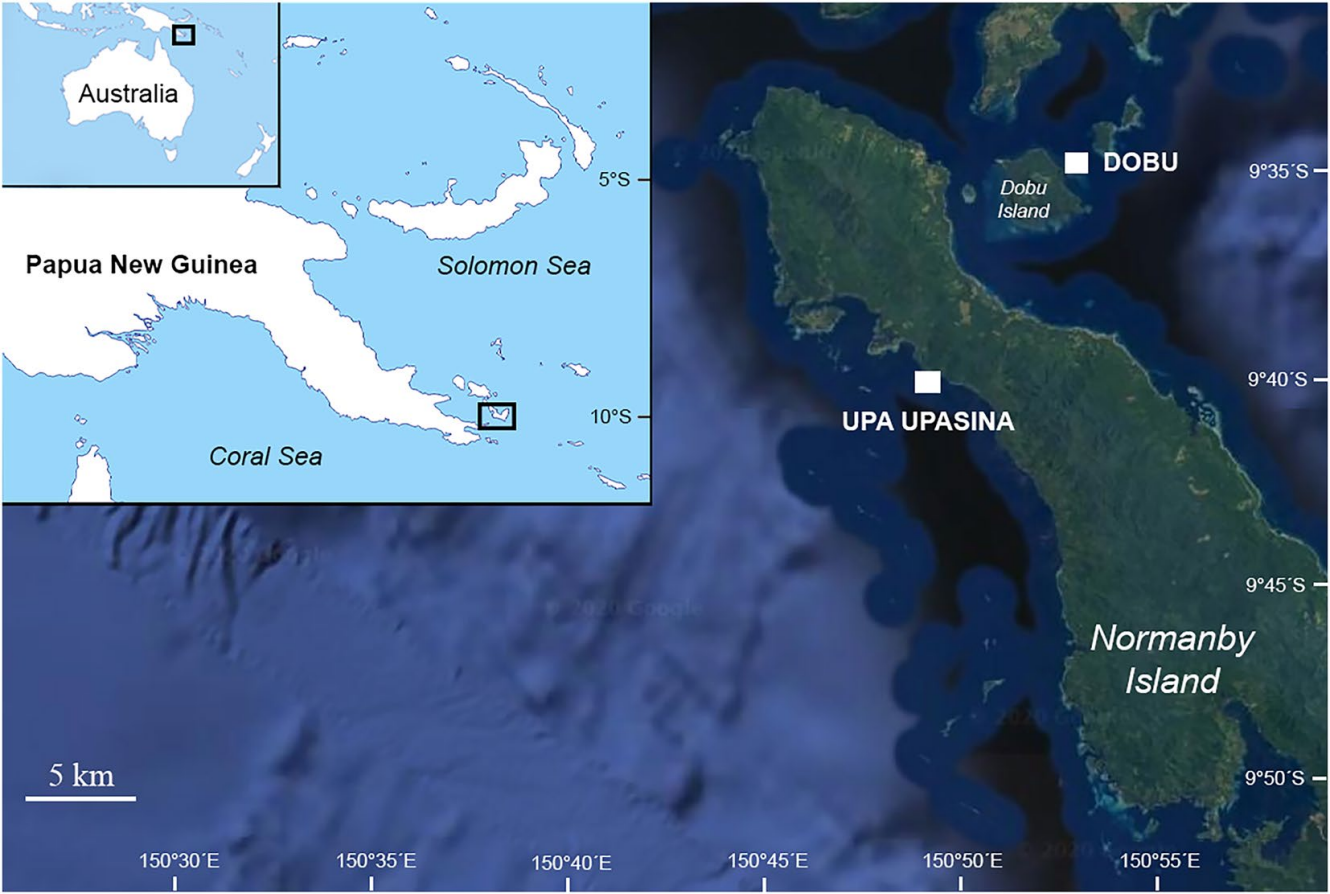
Maps of Papua New Guinea and the two study locations (Dobu and Upa Upasina) on Normanby and Dobu Islands, Milne Bay Province. This Figure was created using Adobe Photoshop CC 2018 (https://www.adobe.com/products/photoshop.html) with imagery from Google Earth (V 9.140.0.4. Eye alt 45 km. SIO, NOAA, U.S. Navy, NGA, GEBCO. TerraMetrix 2020, Digital Globe 2020. https://earth.google.com. 10 December 2020). The inset map was made modifying an image freely available at the following website: https://d-maps.com/carte.php?num_car=3336&lang=en.

The study was conducted at two shallow-water (1–5 m) volcanic CO_2_ seeps at ambient temperature and adjacent control sites at Milne Bay Province, PNG, namely Dobu and Upa Upasina (Fig. 1). Almost pure CO_2_ (~ 99%) has been streaming from the seabed for an unknown period of time (confirmed for approximately 70 years, but likely much longer)^15^, resulting in localized acidified conditions. Environmental parameters (measured: pH, dissolved inorganic carbon, total alkalinity, salinity, and temperature; calculated with CO2SYS software: pCO_2_ and aragonite saturation state) were obtained across a 4-year period (2010–2013) at 1–5 m depth in both control and seep sites^15,24^. Two-cm coral fragments, which corresponds to approximately a 1.5-year growth increment in all the investigated species^15,26,27^, were collected at 1–5 m depth from adult colonies of *P. damicornis*, *G. fascicularis*, *A. millepora*, and massive *Porites* at control and seep sites in August 2010 (N = 6–15 fragments per site, each fragment from a different colony)^28^. Tissue from the coral fragments was totally removed using established protocols applied in previous studies on corals which include immersing the samples in a solution of 10% commercial bleach for 3 days and drying them for 24 h at a maximum temperature of 40°C^29–32^. Dried fragments were kept in codified Eppendorf tubes prior to skeletal measurements.

### Skeletal porosity, bulk density, and micro-density determination

The skeletal porosity, bulk density, and micro-density of the 192 fragments from the control and seep sites at Dobu and at Upa Upasina were obtained as follows. After determining the dry mass, the fragments were placed inside a drying chamber connected to a vacuum pump to evacuate air and water from the pores, a necessary step in order to allow effective saturation of the samples in the followig phase. After 3 h, distilled water was gently introduced to fully saturate the samples which were then weighed in air to determine the saturated weight. Buoyant weight was then measured with a hydrostatic balance (Ohaus Explorer Pro balance ± 0.0001 g) equipped with a density determination kit and used to calculate porosity, bulk density, and micro-density by means of standard calculations (details in Supplementary Methods)^29^.

### Time-domain nuclear magnetic resonance for pore size distribution determination

To investigate the distribution of pore-size classes, through the analysis of the NMR transverse relaxation time *T*_2_ distributions from control and seep site (details in Supplementary Methods), coral fragments still fully saturated with water, were placed on a wet paper to dry the excess of water on their surface. Then, every fragment was put inside a glass tube, sealed and immediately inserted into the magnetic field to be subjected to TD-NMR measurement. A home-built relaxometer based on an electromagnet JEOL C-60 (magnetic field B_0_ = 0.5 Tesla) with a radiofrequency coil ≈ 8 mm in diameter, and equipped with a Spinmaster portable console (Stelar, Mede, Pavia, Italy) was used. The Carr- Purcell-Meiboom-Gill (CPMG) sequence with 200 µs echo time was used to acquire for each specimen the transverse relaxation curve. The measured multi-exponential relaxation curves, affected by unavoidable measurement noise, were transformed into distributions of the transverse relaxation time *T*_2_ by the algorithm UPEN (Uniform-Penalty inversion algorithm)^33^, implemented in the UpenWin software^34^. The ratio between the signal under a particular portion of the *T*_2_ distribution and the total acquired signal will correspond to the ratio of the volume of the pores with a particular pore size to the total pore volume. TD-NMR measurements were performed on 2 fragments per site per species for *A. millepora*, massive *Porites*, and *G. fascicularis* and on all available fragments of *P. damicornis* (a total of 60 fragments). The *T*_2_ distributions showed a cut-off at 3 ms allowing to divide the pores containing water into two classes, distinguishing the smaller pores (smaller volumes, estimated pore sizes < 1 µm) from the remaining larger ones (larger volumes, estimated pore sizes > 1 µm). For the sake of simplicity, the two classes were named micro-scale and macro-scale pores^13,35^.

### Thermogravimetric analysis for organic matrix content determination

To determine the intraskeletal organic matrix and water content, thermal gravimetric measurements were performed using a TA Instruments thermobalance model SDT-Q600 with 0.1 µg of balance sensitivity. Powdered subsamples (5 to 10 mg), held in alumina pans, were heated under a linear gradient from ambient (ca. 20 °C) up to 600 °C with an isotherm at 120 °C for 5 min to remove the adsorbed water; heating rate: 10 °C/min under an N_2_ atmosphere, with flux fixed to 100 ml/min. Two main weight loss regimes were identified: a first one in a range around 125–250° C (related to the loss of structured water molecules) followed by another thermal region between 250 and 470 °C (generally associated with organic matrix pyrolysis)^36^. A total of 32 fragments from the control and seep sites at Dobu (10 for each site) and at Upa Upasina (6 for each site) were analyzed.

### Synchrotron high-resolution X-ray powder diffraction

To determine crystallite parameters, coral fragments were measured at the ID22 beamline of the European Synchrotron Radiation Facility (Grenoble, France) using a wavelength of 0.4 Å (details in Supplementary Methods). Subsamples of the fine powder were loaded into borosilicate glass capillaries of 0.7–1 mm in diameter and measured at room temperature, and again after ex-situ heating at 300 °C for 2 h, to remove the organic matrix effects on the strain^37^. Rietveld refinement was used to calculate the unit-cell parameters from the diffraction pattern profiles. The line profile analysis was applied to a specific diffraction peak to obtain the coherence length (nm) along various crystallographic directions, which was achieved by fitting the profile to a Voigt function and deconvoluting the Lorentzian and Gaussian widths. Analyses were conducted on fragments of *P. damicornis* from Upa Upasina in the control (N = 3) and seep (N = 3) site.

### Statistical analyses

Permutation multivariate analysis of variance (PERMANOVA) was perfomed using PRIMER v6^38^ and based on Euclidean distances (999 permutation) to test for (1) variations of environmental parameters amongst locations and sites; (2) variations of skeletal parameters amongst locations, sites, and species. When the main tests revealed statistical differences (*P* < 0.05), PERMANOVA pairwise comparisons were carried out. The BEST routine in PRIMER v6 (999 permutations) was carried out to check for auto-correlated environmental variables, thus obtaining the minimum subset of variables that may explain differences in environmental conditions amongst locations, sites and seasons (i.e., Spring included data collected in April and May; Winter included data collected in January and December). Organic matrix related strain and crystallite size in *P. damicornis* were compared between control and seep sites using the non-parametric Mann–Whitney U-test, due to deviations from parametric t-test assumption (Normality: Shapiro–Wilk's test; equal variance: Levene’s test). This statistical analysis was performed using SPSS 20.0. Data visualization and graphics were obtained with the ggplot2 package in R^39^. Statistical differences were accepted when *P* < 0.05.

## Results

### Environmental parameters

The values of the environmental parameters collected at control and seep sites in Dobu and Upa Upasina over a 3-year period are summarized in Fig. S1. Briefly, pH and pCO_2_ across both seep sites averaged 7.72 ± 0.23 (SD) and 1133 ± 1161 µatm, while at the control sites it averaged 7.93 ± 0.10 and 518 ± 250 µatm, respectively. The complete dataset of environmental parameters (Fig. S1) was analyzed to test for differences between sampling locations, and between control *vs* seep sites within each location. Effects of seasonality were also considered. PERMANOVA analyses showed that environmental conditions were different between locations and sites and that seasons did not differ significantly (Supplementary Table S1 and Fig. S1). PERMANOVA pair-wise comparisons showed that within each of the two locations control and seep sites were significantly different (Dobu: t = 3.127, *P* = 0.001; Upa Upasina: t = 2.547, *P* = 0.001). The two control sites also differed between the two locations (t = 2.112, *P* = 0.002), while seep sites were similar (t = 1.244, *P* = 0.154). The BEST routine revealed that pCO_2_ and Ω_AR_ were strongly autocorrelated with the other environmental parameters (Rho = 0.995, *P* = 0.001) and were therefore excluded from the following PERMANOVA analysis. PERMANOVA analyses on single environmental parameters showed that pH and total alkalinity were significantly different between control and seep sites, while temperature was significantly different between locations (Table 1 and Supplementary Table S1). DIC was significantly different between controls of Upa Upasina and Dobu (t = 6.137, *P* = 0.001) while at the seep sites DIC was homogeneous (t = 0.061, *P* = 0.962). Salinity was unchanged between either locations or sites (Table 1 and Supplementary Table S1).

**Table 1:** Means and standard deviation (in parenthesis) of the investigated environmental parameters in seep and control sites in Dobu and Upa Upasina.

Location	Dobu		Upa Upasina	
Site	Control	Seep	Control	Seep
pH	7.96 (0.04)	7.66 (0.27)	7.91 (0.13)	7.75 (0.19)
	N = 46	N = 130	N = 67	N = 222
	a	b	a	b
DIC	1946 (15)	2106 (90.0)	2082 (38)	2060 (30.0)
	N = 32	N = 30	N = 71	N = 254
	a	b	c	b
TA	2235 (9)	2275 (1)	2252 (21)	2285 (18)
	N = 47	N = 207	N = 71	N = 254
	a	b	a	b
Salinity	34.9 (0.8)	34.7 (0.7)	35.2 (0.8)	34.8 (0.7)
	N = 59	N = 207	N = 71	N = 254
	a	a	a	a
T (°C)	29.1 (1.4)	29.0 (0.7)	30.1 (1.3)	30.2 (0.9)
	N = 59	N = 207	N = 71	N = 242
	a	a	b	b

Lettering indicates significantly different groups (PERMANOVA on single parameters).

*pH*_*TS*_ pH in total scale, *pCO*_*2*_ carbon dioxide partial pressure, *Ω*_*AR*_ aragonite saturation, *DIC* dissolved inorganic carbon, *TA* total alkalinity, *T* seawater temperature, *N* number of measurements.

### Skeletal parameters in corals sampled at the control and seep sites of Dobu and Upa Upasina

**Figure 2:**
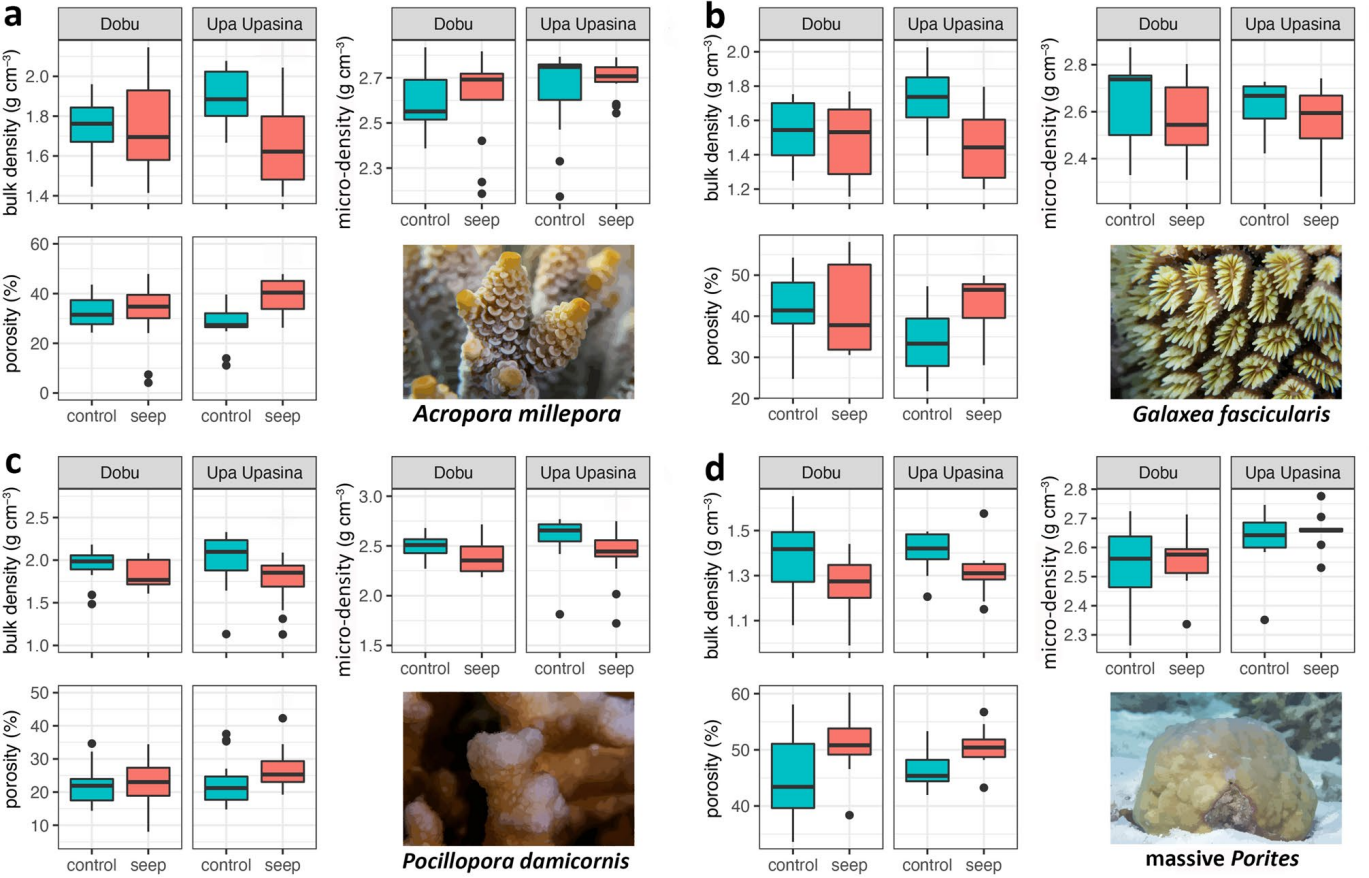
Skeletal parameters micro-density, porosity and bulk density at control (green box plots) and seep sites (pink box plots) in Dobu and Upa Upasina (UPA) for (**a**) *Acropora millepora*, (**b**) *Galaxea fasciularis*, (**c**) *Pocillopora damicornis*, and (**d**) massive *Porites*. The boxes indicate the 25th and 75th percentiles and the line within the boxes mark the medians. Whisker length is equal to 1.5 × interquartile range (IQR). Circles represent outliers. Statistical analyses for these data are reported in Tables 2 and 3. Plots were created with the R package ggplot2^62^. Plots were created with the R package ggplot2^62^. (photographs by co-author FR).

Results for bulk density, micro-density, and porosity are reported in Fig. 2 and in Supplementary Table S2. PERMANOVA analyses indicated significant differences among species in micro-density, porosity, and bulk density (Table 2). Porosity and bulk density were also significantly different between sites (Table 2). For all species, porosity and bulk density were significantly different between control and seep sites at Upa Upasina (t = 4.752, *P* = 0.001 and t = 5.864, *P* = 0.001, respectively), with higher porosity and lower bulk density at the seep site compared to the control (Fig. 2). Bulk density was significantly lower at the seep site compared to the control also at Dobu (t = 2.675, *P* = 0.010; Fig. 2). Micro-density showed a significant interaction between the factor Site and Species (Table 2); indeed micro-density values assessed in *P. damicornis* were significantly lower at the seep site compared to the control at both locations (Fig. 2; Table 3).

**Table 2:** Results of the PERMANOVA analysis for porosity, bulk density, and micro-density in the control and seep sites at Dobu and Upa Upasina for *Acropora millepora*, *Galaxea fasciularis*, *Pocillopora damicornis*, and massive *Porites*.

	df	Porosity		Bulk density		Micro-density	
		Pseudo-F	*P*	Pseudo-F	*P*	Pseudo-F	*P*
Sp	3	61.541	**0.001**	78.244	**0.001**	10.096	**0.001**
Lo	1	0.034	0.853	3.791	0.054	8.732	**0.007**
Si(Lo)	2	10.374	**0.001**	20.775	**0.001**	2.645	0.073
SpxLo	3	0.276	0.841	0.174	0.922	1.487	0.223
SpxSi(Lo)	6	0.850	0.546	1.211	0.327	3.535	**0.006**

Significant values are reported in bold.

*Sp* species, *Lo* location, *Si* site.

**Table 3:** PERMANOVA pairwise comparisons for micro-density based between Control and Seep sites within locations (Dobu and Upa Upasina) for the four investigated species.

	Upa Upasina		Dobu	
	t	*P*	t	*P*
*Acropora milepora*	1.002	0.375	0.815	0.440
*Galxea fascicularis*	1.092	0.291	0.802	0.424
massive *Porites*	0.813	0.488	0.476	0.650
*Pocillopora damicornis*	4.787	**0.002**	3.615	**0.003**

The pairwise test was conducted only for micro-density based on the significant interaction between Site and Species (Table 2). Significant values are reported in bold.

**Figure 3:**
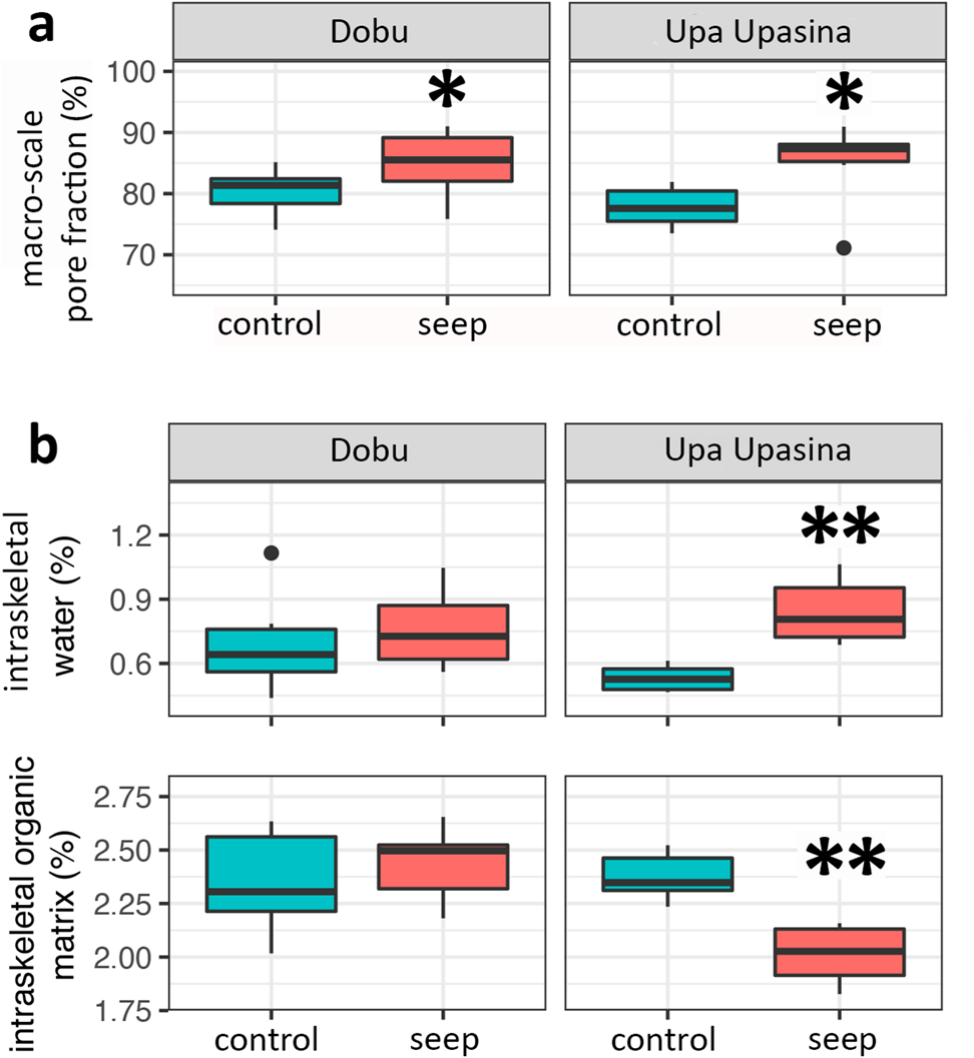
Macro-scale pore volume fraction (in the figure simply macro pore fraction) and intraskeletal OM and water content for *P. damicornis* from control and seep sites at Dobu and Upa Upasina. (**a**) Time Domain-Nuclear Magnetic Resonance measurement of macro-scale pore volume fraction. (**b**) Thermogravimetric Analysis of microscale parameters, namely intraskeletal organic matrix (OM), intraskeletal water content, and total (the sum of OM and water). The box indicates the 25th and 75th percentiles and the line within the box marks the median. Whisker length is equal to 1.5 × interquartile range (IQR). Circles represent outliers. Significant differences between control and seep sites are represented by asterisks, **P < 0.01, *P < 0.05. Plots were created with the R package ggplot2^62^.

Micro-density changes in *P. damicornis* were further explored both statistically and through additional macroscale and microscale skeletal analyses. Specifically, Time-Domain Nuclear Magnetic Resonance (TD-NMR), Thermogravimetric Analysis (TGA), and synchrotron high-resolution powder X-ray diffraction (HRPXRD) analyses were performed. TD-NMR measurements were performed on two fragments for all species to have a general overview of the *T*_2_ distributions (Supplementary Fig. S2). Further analyses were conducted on all available fragments of *P. damicornis* to quantify macro-scale pore volume fraction. PERMANOVA analyses showed that macro-scale pore volume fraction was significantly higher at the seep site compared to the control in both locations (Upa Upasina: t = 2.126, *P* = 0.041; Dobu: t = 2.549, *P* = 0.028; Fig. 3 and Supplementary Table S3). The intraskeletal organic matrix (OM; t = 4.856, *P* = 0.004) and water content (t = 4.891, *P* = 0.001) were significantly different between Sites only at Upa Upasina (Table 4; Fig. 3; Supplementary Table S4). In particular, the former showed lower values at the seep site compared to control, while the latter showed the opposite trend (Fig. 3).The intraskeletal OM content was significantly different also among locations (Table 4).

**Table 4:** Results of the PERMANOVA analysis for intraskeletal water and organic matrix (OM) content and macro-scale pore fraction volume in *Pocillopora damicornis* in control and seep sites at Dobu and Upa Upasina.

Factor	df	Pseudo-F	*P*
**Intraskeletal water content**			
Lo	1	0.279	0.608
Si(Lo)	2	6.831	**0.005**
**Intraskeletal organic matrix content**			
Lo	1	12.318	**0.001**
Si(Lo)	2	7.164	**0.002**
**Macro-scale pore volume fraction**			
Lo	1	0.775	0.380
Si(Lo)	2	5.715	**0.012**

*Lo* location, *Si* site.

Three *P. damicornis* skeletal fragments from the control and from the seep sites in Upa Upasina were analysed by HRPXRD. All HRPXRD patterns were well indexed as aragonite and no additional diffraction peaks were detected. Then, the peaks were refined using the Rietveld method^40^ and lattice parameters and strain (Supplementary Table S5), and microstructural data^41^, crystallite size, and microstrain (Supplementary Table S6), were calculated. No significant differences were found between the control and seep site. To test the influence of the OM on the mineral strain, ex-situ heat treatments prior the HRPXRD measurements, which remove the effect of the OM on the strain^37^, were performed. The data showed that OM induced a positive strain on *a*- and *c*-axis and a negative one on the *b*-axis, but no significant differences were found between the control and seep site (Supplementary Table S5). We also measured crystallite size after the thermal annealing together with the transition to calcite (Supplementary Tables S5 and S6). These latter parameters did not show any significant difference between the control and seep sites.

## Discussion

In the past decades, significant efforts have been made to quantify the ecological effects of ongoing ocean acidification (OA) in tropical regions. However, assessing the effects of OA on reef-building corals poses major challenges because multiple environmental changes, including ocean warming, are co-occurring with OA, impacting coral growth^42,43^. This study investigated the effects of long-term exposure to elevated CO_2_ on skeletal properties in tropical zooxanthellate corals naturally living at CO_2_ vents.

Similar to Mediterranean^13^ and other tropical coral species^44^, increased porosity and decreased bulk density was observed at the seep sites compared to the control sites, with some species showing more marked trends than others, in agreement with a general decreasing trend of net calcification rates at relatively low pH conditions resembling IPCC projections^15,45^. A 2-year field transplant experiment conducted on *Porites astreoides*, *Siderastrea siderea* and *Porites porites* at low pH submarine springs in the Yucatán peninsula (Mexico) showed species-specific OA-related vulnerability in calcification rates which may be linked to differential growth rates, with fast-growing corals being likely more sensitive to low carbonate ion availability^46^. Species-specific sensitivities to OA also depend on its impacts on chemistry within the calcifying fluid^47,48^ and/or in the diverse use of metabolic reserves^49^. Moreover, different populations of the same species might display variable responses to OA, as highlighted for instance by the intra-specific variability displayed by calcification rates of *Acropora digitifera* from two distinct locations after exposure to acidified conditions in aquaria^50^. All these aspects could contribute to explain the variability observed by the different species in the two locations considered in the current study.

Micro-density showed lower values at the seep sites compared to the control sites of both locations only in *P. damicornis.* Micro-density, which represents the mass per unit volume of the biogenic calcium carbonate composing the skeleton^51^, depends on the mineral composition of the skeleton and on intraskeletal organic matrix (OM) and water content^29^. The evaluation of additional macro- and micro- scale parameters performed in this species also revealed an increase in macro-scale pore volume fraction and intraskeletal water content and a decrease in OM, and eventually strong linked water^36^. In particular, the observed increase of intraskeletal water content at the seep site can partially justify the observed decrease in skeletal micro-density. According to literature, the observed decrease of skeletal micro-density could also stem from a variation in chemistry and micro-architecture of the skeleton^52^, the presence of occluded nano-porosity^13^, and the presence of amorphous calcium carbonate^53^. Changes in OM and water content with pH reduction have been previously reported, showing either an increase in the tropical *Stylophora pistillata* kept in aquaria at pH 7.2 for approximately 1 year^36,44^, or no variation in the temperate *Balanophyllia europaea* naturally living along a CO_2_ vent at average seawater pH 7.7^32^. Moreover, *P. damicornis* exposed to pH 7.8, 7.4 and 7.2 in aquaria for 3 weeks showed a 4 to 70-fold up-regulation of genes encoding skeleton organic matrix proteins at all pH treatments^54^. In these studies, the observed up-regulation of genes linked to OM proteins and the increase in OM content was hypothesized to promote calcification under less favorable acidified conditions. Thus, the observed decline in OM in *P. damicornis* in the current study suggests a possible decline in net calcification rates at the seep site, which is in agreement with the observed increase in skeletal porosity. However, considering the natural setting in which the study was performed, we cannot exclude the influence of other covarying environmental factors in determining the observed responses (e.g., turbidity, light availability, organic/inorganic nutrient availability, feeding)^55–58^.

The decrease of intra-skeletal OM content in samples from seep sites was not associated with a significant change in strain, micro-strain, or crystallite size. These observations may indicate that the amount of intra-crystallite OM does not change since the crystallite sizes after the thermal annealing are the same for samples from the control and the seep sites. Thus, the observed decrease in OM is likely associated with a decrease in the inter-crystallite OM. In addition, the stability of aragonite through the transition to calcite and the lattice parameters of the calcite formed after thermal annealing did not show a significant difference between control and seep samples. The crystallographic features of aragonite from coral skeletons have been previously investigated^59^. The reef building coral *Stylophora pistillata* grown in aquaria under different experimental seawater acidification (pH 8.2, 7.6, and 7.3) showed anisotropic distortions of aragonite lattice parameters and a reduction of the crystallite sizes under acidified conditions^36^. In the presented study, these parameters were unaffected by decreasing pH, suggesting that biological control over calcification does not change at the nanoscale, as reported for *B. europaea*^13^. The fact that different species were used, but most of all that *S. pistillata* was exposed for 1 year (short-term acclimation) while in the current study species were exposed to acidified conditions for generations (life-long acclimatization) likely accounts for these discrepancies. The calcite phase obtained by annealing of coral samples has similar lattice parameters in samples from the control and seep sites. These parameters, when compared with those of synthetic calcite^60^, did not show differences. A different behavior was observed for calcite obtained from *Desmophyllum* and *Favia* aragonitic skeletons, which showed different strain compared with geological or synthetic calcite^59^.

## Conclusions

This multi-species study showed a common phenotypic response among four zooxanthellate corals which displayed a more porous skeletal phenotype under OA. Additionally, these skeletal macromorphological adjustments led to decreased micro-density in *P. damicornis* but did not affect the measured crystallite features, suggesting that the fundamental structural components produced by the biomineralization process might be substantially unaffected by increased acidification^13,61^. Nonetheless, the porous phenotype here described may render structurally complex and massive corals more vulnerable to damage and bio-erosion under climate change, which in the future may lead to a weakening of the reef framework and subsequent degradation of the complex coral reef ecosystem.

## Supplementary Information

Supplementary Information.

## Data Availability

The datasets generated during and/or analysed during the current study are available from the corresponding author on reasonable request.
