# Supplementary material for: Coral micro- and macro-morphological skeletal properties in response to life-long acclimatization at CO2 vents in Papua New Guinea
Source: Sci Rep. 2021 Oct 7;11:19927. doi: 10.1038/s41598-021-98976-9 (PMC8497495; doi:10.1038/s41598-021-98976-9)
Supplement: Supplementary file 1 — Supplementary Information. [file 41598_2021_98976_MOESM1_ESM.docx]

**SUPPLEMENTARY INFORMATION**

**Coral micro- and macro-morphological skeletal properties in response to life-long acclimatization at CO_2_ vents in Papua New Guinea**

Fiorella Prada, Leonardo Brizi, Silvia Franzellitti, Stefano Mengoli, Simona Fermani, Iryna Polishchuk, Nicola Baraldi, Francesco Ricci, Quinzia Palazzo, Erik Caroselli, Boaz Pokroy, Loris Giorgini, Zvy Dubinsky, Paola Fantazzini, Giuseppe Falini, Stefano Goffredo, Katharina E. Fabricius

**Supplementary Methods**

***Skeletal Parameters determination***

Skeletal parameters were obtained by applying the buoyant weight technique from the following measurements: density of the fluid medium (ρ); dry mass of the fragment (DW); buoyant weight of the fragment (BW = weight of the fragment minus weight of the water displaced by it); and SW (saturated weight of the fragment = weight of the fragment plus weight of the water enclosed in its volume). These measurements were used to calculate: V_MATRIX_ (matrix volume = volume of the fragment, not including the volume of its pores); V_PORES_ (pore volume = volume of the pores in the fragment); and V_TOT_ (bulk volume = total volume of the fragment including its pores). The skeletal parameters of the samples were calculated as follows: the micro-density (ratio of DW to V_MATRIX_); the bulk density (ratio DW to V_TOT_); and the porosity (ratio V_PORES_ to V_TOT_).

Definitions:

Micro-density = intended as mass per unit volume of the mineral and intraskeletal organic matrix and water content composing the skeleton, which cannot exceed 2.94 mg/mm^−3^ which is the density of pure aragonite ^1^.

Bulk density = defined as the skeletal dry mass divided by total skeletal volume which includes the skeletal voids

Porosity = defined as the percentage of enclosed volume occupied by pores connected with the external surface compared to the total skeletal volume. Pores inside the biomineral that are not connected to the external surface (occluded pores) are not measured.

***Time-Domain Nuclear Magnetic Resonance for pore size distribution determination***

TD-NMR, and in particular magnetic resonance relaxometry of water ^1^H nuclei, has been validated as a useful tool for analyzing internally connected skeletal porosity in Mediterranean scleractinian corals ^2^. It provides several advantages compared to other methods used for the estimation of pore sizes distribution as it is a non-destructive and non-invasive technique which allows preserving intact specimens for further analyses. TD-NMR has been theoretically established for different porous materials, from silica glass, porcelain samples and sedimentary rocks to biological cells ^3^ and bone tissue ^4^. In this study, the samples, saturated with distilled water, were placed in a static magnetic field that polarized the ^1^H nuclear spins, inducing a nuclear magnetization along the field direction. The process that involves the return of the magnetization vector to equilibrium, after a radio-frequency perturbation, is called NMR relaxation. The evolution of the magnetization vector components has a multi-exponential trend characterized by time constants named *T*_1_ (relaxation time of the longitudinal component) and *T*_2_ (relaxation time of the transverse component). In this study, only the relaxation of the transverse magnetization component was investigated.

In porous media saturated by water, under the assumptions that diffusion of water molecules is fast enough to maintain the nuclear magnetization constant over the pore volume (V_PORES_) before relaxing, including at the surfaces (S), the measured relaxation rate (1/*T*_2_)_observed_ is increased compared to the bulk rate by the amount ρ S/V_PORES_ (where ρ is the surface relaxivity, with the physical dimensions of a velocity, is a constant that depends on the material) following the equation:

(1/ *T*_2_)_observed_ = ρ S/V_PORES_ + 1/*T*_2bulk_

where T_2bulk_ is the relaxation time of the unconfined fluid. Water confined in real porous media shows a distribution of relaxation times that can cover several orders of magnitude, reflecting a wide distribution of local S/V_PORES_ values.

The total NMR signal (SNMR), represented by the area below each *T_2_* distribution, is proportional to the volume of water saturating the pore-space volume V_PORES_. The distribution can be divided in different classes, depending on the shape of the distribution, with shorter relaxation times corresponding to smaller pores.

***Synchrotron high-resolution X-ray powder diffraction***

The measurements were performed at beamline ID22 of the European Synchrotron Radiation Facility (ESRF, Grenoble, France) using a radiation with wavelength of 0.4 Å. This beam line uses a highly collimated and monochromatic beam to perform powder diffraction in the Laue setting. The beam passes through the sample and diffracts, to be collected on the opposite side by a set of 9 synchronized detectors, mounted 2.2° apart. The intensity of the diffractions is integrated over all detectors to produce high-resolution diffraction patterns. Instrument calibration and wavelength refinement have been performed with silicon standard NIST 640c.

**Supplementary References**

1. Bucher, D. J., Harriott, V. J. & Roberts, L. G. Skeletal micro-density, porosity and bulk density of acroporid corals. *J. Exp. Mar. Bio. Ecol.* **228**, 117–136 (1998).

2. Fantazzini, P. *et al.* Gains and losses of coral skeletal porosity changes with ocean acidification acclimation. *Nat. Commun.* **6**, 7785 (2015).

3. Brizi, L. *et al.* Water compartmentalization, cell viability and morphology changes monitored under stress by 1 H-NMR relaxometry and phase contrast optical microscopy. *J. Phys. D. Appl. Phys.* **48**, 415401 (2015).

4. Brizi, L. *et al.* Bone volume-to-total volume ratio measured in trabecular bone by single-sided NMR devices. *Magn. Reson. Med.* **79**, 501–510 (2018).

5. Wickham, H. *ggplot2*. (Springer International Publishing, 2016). doi:10.1007/978-3-319-24277-4

6. Bortolotti, F., Brown, R. & Fantazzini, P. UpenWin: a software for inversion of multiexponential decay data. Windows system Alma Mater Studiorum—Università di Bologna. (2012).

7. Anderson, M. J. PERMANOVA: a FORTRAN computer program for permutational multivariate analysis of variance. Department of Statisitics. *Dep. Stat.* (2005).

**Supplementary Figures**

**
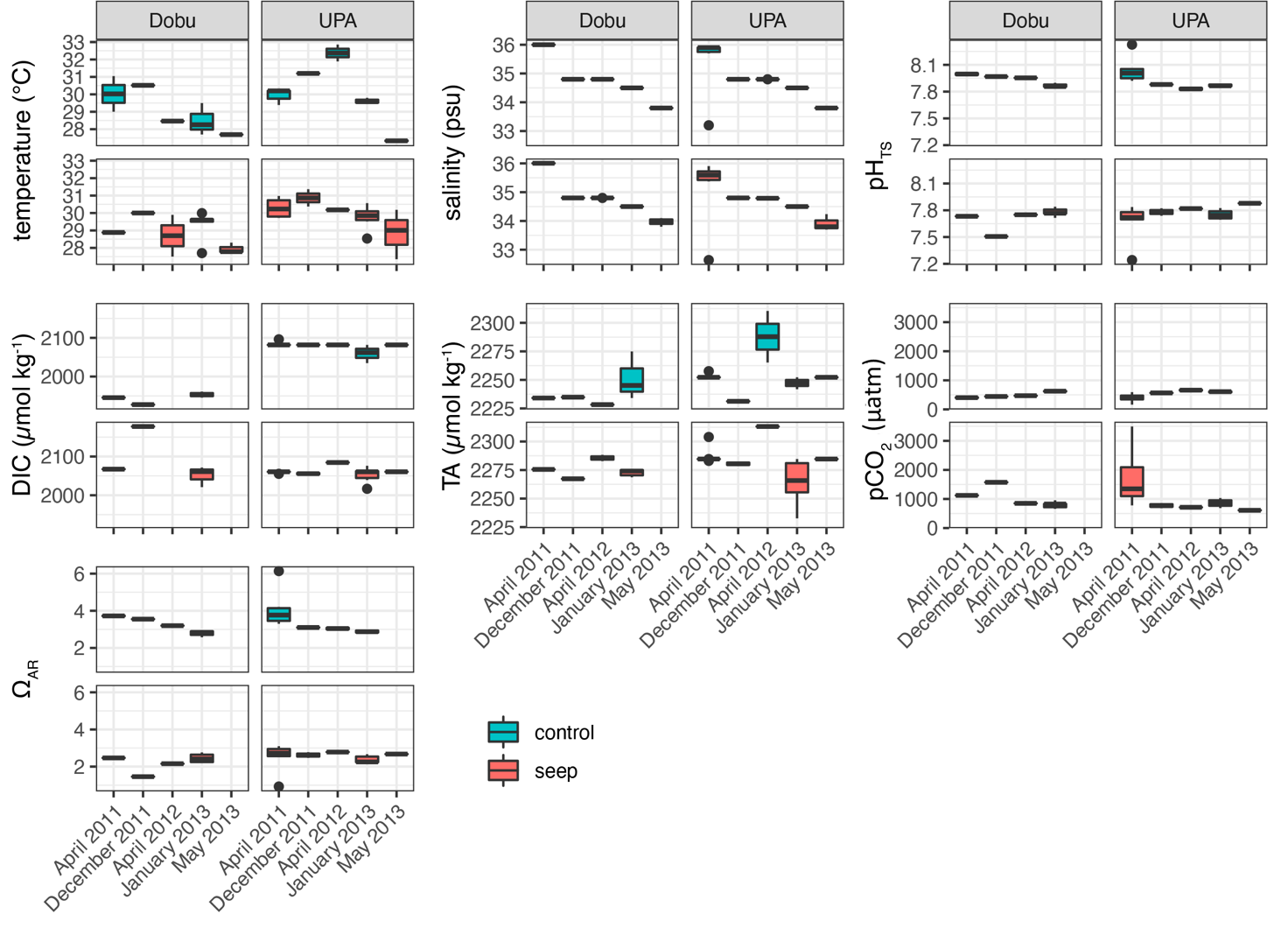
**

**Figure S1.** Environmental parameters at control (green box plots) and seep sites (pink box plots) in Dobu and Upa Upasina (UPA) over a 4-year period (2010-2013). The box indicates the 25^th^ and 75^th^ percentiles and the line within the box marks the median. Whisker length is equal to 1.5 $\times$ interquartile range (IQR). Dots represent outliers. Statistical analyses for these data are reported in Supplementary Table 1. Plots were created with the R package ggplot2^5^.

**
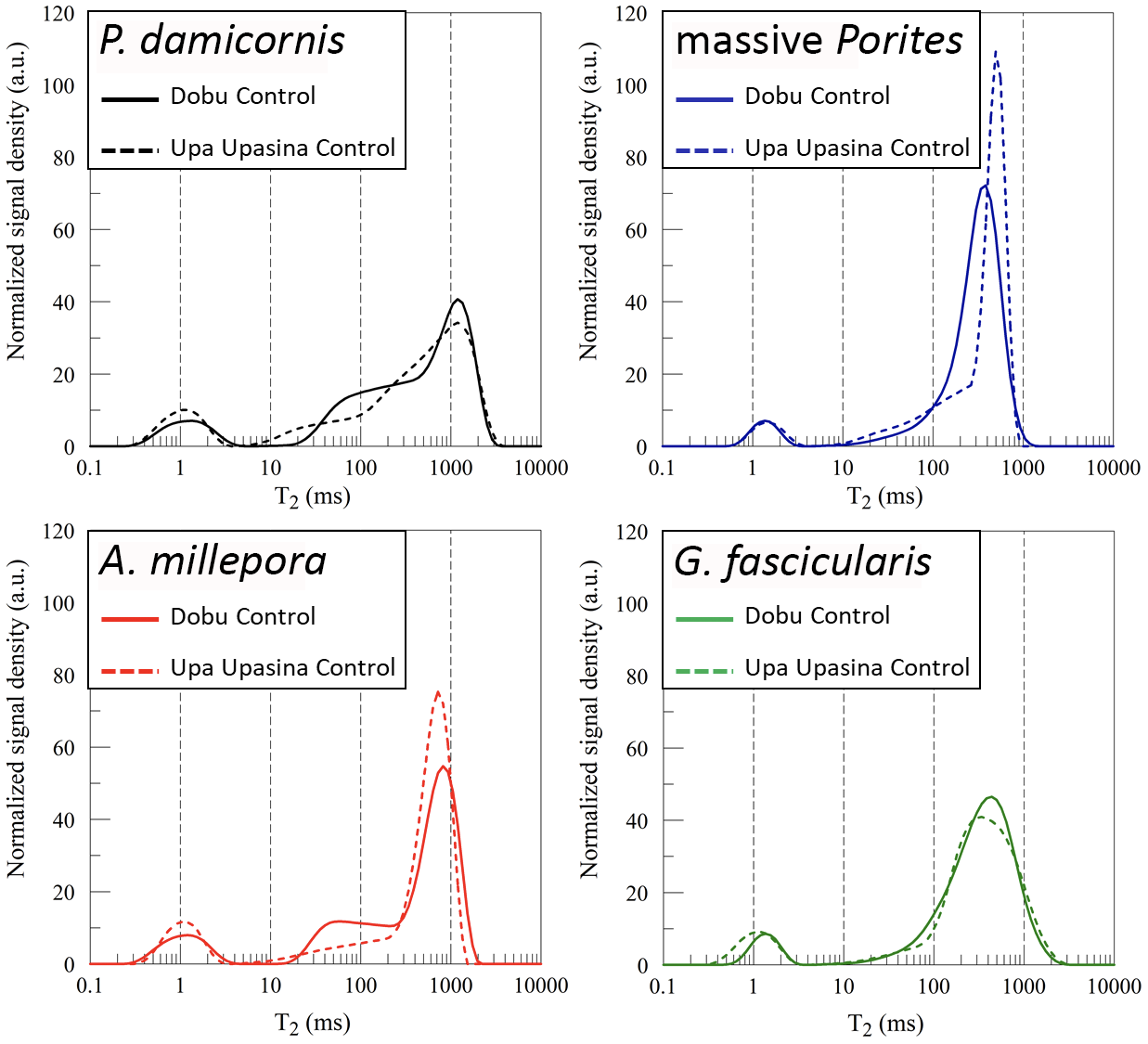
**

**Figure S2.** Representative *T*_2_ distributions for *P. damicornis,* massive *Porites, A. millepora,* and *G. fascicularis* at Dobu and Upa Upasina in control sites. The area below each *T_2_* distribution is proportional to the amount of water saturating the pore space and consequently to the volume of the connected pore space. All the distributions show two classes of pores separated by the cut-off at ~3 ms, called for the sake of simplicity micro-scale and macro-scale pores. Plots were created with the UpenWin software^6^.

**Supplementary Tables**

**Table S1**. Results of the PERMANOVA analysis for the environmental dataset (see Fig. S1) and for pH, DIC, Total Alkalinity, salinity, and temperature (based on the BEST analysis) in control and seep sites at Dobu and Upa Upasina. Significant values are reported in bold. df: degrees of freedom; Pseudo-F: F value by permutation ^7^; P: significance of pseudo-F

|  |  | Whole dataset |  | pH | | DIC | | Total Alkalinity | | Salinity | | Temperature | |
| --- | --- | --- | --- | --- | --- | --- | --- | --- | --- | --- | --- | --- | --- |
| Factor | df | Pseudo-F | P | Pseudo-F | P | Pseudo-F | P | Pseudo-F | P | Pseudo-F | P | Pseudo-F | P |
| Season (Se) | 1 | 1.898 | 0.114 | 1.525 | 0.255 | 0.743 | 0.409 | 3.438 | 0.079 | 1.538 | 0.243 | 0.806 | 0.392 |
| Location (Lc) | 1 | 4.443 | **0.005** | 0.106 | 0.747 | 27.645 | **0.001** | 2.814 | 0.107 | 0.000 | 0.994 | 8.129 | **0.011** |
| Site(Si) | 1 | 11.970 | **0.001** | 19.335 | **0.001** | 12.029 | **0.001** | 43.038 | **0.001** | 0.770 | 0.386 | 0.065 | 0.803 |
| SexLc | 1 | 0.896 | 0.459 | 0.142 | 0.715 | 0.489 | 0.499 | 6.412 | **0.012** | 0.002 | 0.959 | 0.271 | 0.600 |
| SexSi | 1 | 0.851 | 0.476 | 0.339 | 0.553 | 2.625 | 0.115 | 0.540 | 0.460 | 0.680 | 0.411 | 0.745 | 0.395 |
| LcxSi | 1 | 2.770 | **0.028** | 0.061 | 0.817 | 28.388 | **0.001** | 0.027 | 0.874 | 0.140 | 0.702 | 0.023 | 0.870 |
| SexLcxSi | 1 | 0.571 | 0.686 | 0.723 | 0.384 | 1.210 | 0.289 | 0.067 | 0.797 | 0.103 | 0.761 | 0.507 | 0.485 |

**Table S2.** Means and standard deviations (SD) of the skeletal parameters micro-density, porosity and bulk density in seep and control sites for the investigated species.

|  |  | Micro-density  (g cm^-3^) | |  |  |  | Porosity  (%) | |  |  |  | Bulk density  (g cm^-3^) | |  |  |  |
| --- | --- | --- | --- | --- | --- | --- | --- | --- | --- | --- | --- | --- | --- | --- | --- | --- |
| species |  | N | mean | SD |  |  | N | mean | SD |  |  | N | mean | SD |  |  |
|  | **Dobu** | | | | | | | | | | | | | | | |
| *Acropora millepora* | Seep | 14 | 2.62 | 0.19 |  |  | 14 | 32.27 | 12.61 |  |  | 14 | 1.75 | 0.22 |  |  |
|  | Control | 14 | 2.59 | 0.14 |  |  | 14 | 32.13 | 6.44 |  |  | 14 | 1.76 | 0.14 |  |  |
| *Galaxea fascicularis* | Seep | 10 | 2.56 | 0.17 |  |  | 10 | 41.60 | 10.95 |  |  | 10 | 1.49 | 0.23 |  |  |
|  | Control | 6 | 2.64 | 0.21 |  |  | 6 | 41.58 | 10.32 |  |  | 6 | 1.53 | 0.20 |  |  |
| massive *Porites* | Seep | 10 | 2.55 | 0.10 |  |  | 10 | 51.06 | 6.04 |  |  | 10 | 1.25 | 0.14 |  |  |
|  | Control | 10 | 2.53 | 0.15 |  |  | 10 | 44.77 | 7.73 |  |  | 10 | 1.39 | 0.17 |  |  |
| *Pocillopora damicornis* | Seep | 15 | 2.39 | 0.17 |  |  | 15 | 22.42 | 7.14 |  |  | 15 | 1.85 | 0.16 |  |  |
|  | Control | 15 | 2.49 | 0.11 |  |  | 15 | 22.17 | 6.32 |  |  | 15 | 1.94 | 0.19 |  |  |
|  | **Upa Upasina** | | | | | | | | | | | | | | | |
| *Acropora millepora* | Seep | 15 | 2.70 | 0.07 |  |  | 15 | 38.71 | 7.18 |  |  | 15 | 1.65 | 0.19 |  |  |
|  | Control | 15 | 2.65 | 0.19 |  |  | 15 | 27.98 | 7.65 |  |  | 15 | 1.90 | 0.14 |  |  |
| *Galaxea fascicularis* | Seep | 8 | 2.56 | 0.16 |  |  | 8 | 42.88 | 7.87 |  |  | 8 | 1.46 | 0.22 |  |  |
|  | Control | 11 | 2.64 | 0.10 |  |  | 10 | 34.35 | 8.46 |  |  | 10 | 1.72 | 0.22 |  |  |
| massive *Porites* | Seep | 9 | 2.66 | 0.07 |  |  | 9 | 50.57 | 3.86 |  |  | 9 | 1.31 | 0.12 |  |  |
|  | Control | 10 | 2.62 | 0.11 |  |  | 10 | 46.41 | 3.51 |  |  | 10 | 1.41 | 0.09 |  |  |
| *Pocillopora damicornis* | Seep | 15 | 2.41 | 0.26 |  |  | 15 | 27.01 | 6.06 |  |  | 15 | 1.77 | 0.28 |  |  |
|  | Control | 15 | 2.59 | 0.23 |  |  | 15 | 22.99 | 7.47 |  |  | 15 | 2.00 | 0.32 |  |  |

**Table S3.** Macro-scale pore volume fraction for *P. damicornis* obtained by TD-NMR analysis in control and seep sites in the two locations. The mean, standard deviations, number of corals examined and p value by the non-parametric test are reported. N is the number of corals examined.

| Location | Site | Mean (%) | SD (%) | N |
| --- | --- | --- | --- | --- |
| Dobu | control | 81.3 | 3.9 | 15 |
|  | seep | 83.7 | 5.0 | 15 |
| Upa Upasina | control | 78.9 | 4.9 | 15 |
|  | seep | 84.4 | 6.7 | 15 |

**Table S4.** Results of thermo-gravimetric analysis on *P. damicornis* samples from Dobu and Upa Upasina.

| Variable | site | N | Mean  % mass loss | SD  % mass loss |
| --- | --- | --- | --- | --- |
| **Dobu** | | | | |
| H_2_O | control | 10 | 0.675 | 0.193 |
|  | seep | 10 | 0.755 | 0.168 |
| OM | control | 10 | 2.364 | 0.213 |
|  | seep | 10 | 2.528 | 0.250 |
| **Upa Upasina** | | | | |
| H_2_O | control | 6 | 0.531 | 0.061 |
|  | seep | 6 | 0.844 | 0.155 |
| OM | control | 6 | 2.376 | 0.113 |
|  | seep | 6 | 2.014 | 0.140 |

**Table S5**. Aragonite crystallographic axes, macrostrain associated to the organic matrix (removed by thermal annealing) and aragonite to calcite transition after thermal annealing (300 °C for 2 hours) of skeletal fragments of *P. damicornis* sampled in control and seep sites at Upa Upasina (N = 3 for control and for seep sites). The statistical significance between control and seep sites by the non-parametric Kruskal-Wallis test is reported in the last row. Values are indicated as means with standard deviation in parenthesis.

|  | aragonite | | | | | | |  | calcite after thermal annealing | | |
| --- | --- | --- | --- | --- | --- | --- | --- | --- | --- | --- | --- |
| Site | crystallographic axis | | |  | macrostrain | | |  | % | crystallographic axis | |
|  | a (Å) | b (Å) | c (Å) |  | Δa | Δb | Δc |  |  | a | c |
| control | 4.96E+00 (2.71E-05) | 7.97E+00 (9.78E-04) | 5.75E+00 (2.82E-04) |  | 5.53E-04  (5.01E-05) | -2.15E-04 (2.27E-04) | 4.94E-04 (1.21E-04) |  | 3.73E+01  (6.03E+00) | 4.9842  (5) | 17.06  (1) |
| seep | 4.96E+00 (9.19E) | 7.97E+00 (3.86E-04) | 5.75E+00 (3.88E-04) |  | 5.10E-04  (5.70E-05) | -2.09E-04 (6.24E-05) | 4.35E-04 (1.40E-04) |  | 3.70E+01  (2.65E+00) | 4.9842  (1) | 17.064  (4) |
| Statistical significance | NS | NS | NS |  | NS | NS | NS |  | NS | NS | NS |

**Table S6**. Crystallite size and microstrain estimation for the planes (111), (021) and (221) of aragonite using peak broadening equations and Rietveld parameters skeletal fragments of *P. damicornis* sampled in control and seep sites at Upa Upasina (N = 3 for control and for seep sites). The statistical significance between control and seep sites by the non-parametric Kruskal-Wallis test is reported in the last column to the right. Values are indicated as means with standard deviation in parenthesis.

|  |  | (111) | | (021) | | (221) | |  |
| --- | --- | --- | --- | --- | --- | --- | --- | --- |
| Site |  | crystallite size (um) | microstrain  (%) | crystallite size (um) | microstrain (%) | crystallite size (um) | microstrain (%) | Statistical significance |
| control | pristine | 2.77E-01  (8.31E-02) | 2.27E-01  (1.23E-02) | 2.48E-01  (9.44E-02) | 1.98E-01  (1.12E-01) | 2.06E-01  (5.23E-02) | 1.652E-01  (2.03E-02) | NS |
| seep |  | 2.74E-01  (4.84E-02) | 2.276E-01  (1.33E-02) | 2.35E-01  (5.66E-02) | 1.71E-01  (8.68E-02) | 2.00E-01  (3.44E-02) | 1.50E-01  (1.95E-02) |  |
| control | after thermal annealing | 1.13E-01  (1.03E-02) | 8.75E-01  (1.86E-02) | 1.08E-01 (3.14E-03) | 8.55E-01 (7.45E-02) | 7.06E-02 (9.94E-03) | 6.08E-01 (2.41E-01) | NS |
| seep |  | 1.04E-01 (7.46E-03) | 5.70E-01  (1.54E-01) | 1.08E-01 (1.29E-02) | 8.21E-01 (1.40E-01) | 7.07E-02 (2.56E-03) | 3.30E-01 (2.27E-02) |  |
